# Bmi-1: A master regulator of head and neck cancer stemness

**DOI:** 10.3389/froh.2023.1080255

**Published:** 2023-01-16

**Authors:** Alexandra E. Herzog, Ritu Somayaji, Jacques E. Nör

**Affiliations:** ^1^ Department of Cariology, Restorative Sciences, Endodontics, University of Michigan School of Dentistry, Ann Arbor, MI, United States; ^2^ Department of Otolaryngology – Head and Neck Surgery, University of Michigan Medical School; Ann Arbor, MI, United States; ^3^ Department of Biomedical Engineering, University of Michigan College of Engineering, Ann Arbor, MI, United States; ^4^ University of Michigan Rogel Cancer Center, Ann Arbor, MI, United States

**Keywords:** head and neck cancer, salivary gland cancer (SGC), cancer stem cell (CSC), self-renewal, bmi-1, therapeutic resistance, head and neck squamos cell carcinoma, targeted therapy

## Abstract

Head and neck cancers are composed of a diverse group of malignancies, many of which exhibit an unacceptably low patient survival, high morbidity and poor treatment outcomes. The cancer stem cell (CSC) hypothesis provides an explanation for the substantial patient morbidity associated with treatment resistance and the high frequency of tumor recurrence/metastasis. Stem cells are a unique population of cells capable of recapitulating a heterogenous organ from a single cell, due to their capacity to self-renew and differentiate into progenitor cells. CSCs share these attributes, in addition to playing a pivotal role in cancer initiation and progression by means of their high tumorigenic potential. CSCs constitute only a small fraction of tumor cells but play a major role in tumor initiation and therapeutic evasion. The shift towards stem-like phenotype fuels many malignant features of a cancer cell and mediates resistance to conventional chemotherapy. Bmi-1 is a master regulator of stem cell self-renewal as part of the polycomb repressive complex 1 (PRC1) and has emerged as a prominent player in cancer stem cell biology. Bmi-1 expression is upregulated in CSCs, which is augmented by tumor-promoting factors and various conventional chemotherapies. Bmi-1^+^ CSCs mediate chemoresistance and metastasis. On the other hand, inhibiting Bmi-1 rescinds CSC function and re-sensitizes cancer cells to chemotherapy. Therefore, elucidating the functional role of Bmi-1 in CSC-mediated cancer progression may unveil an attractive target for mechanism-based, developmental therapeutics. In this review, we discuss the parallels in the role of Bmi-1 in stem cell biology of health and disease and explore how this can be leveraged to advance clinical treatment strategies for head and neck cancer.

## Introduction

Head and neck squamous cell carcinoma (HNSCC) is the sixth most common form of cancer worldwide with over 900,000 new cases and 400,000 deaths annually (1). HNSCC incidence is strongly correlated with alcohol and tobacco use, as well as human papillomavirus (HPV) infection. While there has been a marked decrease in HNSCC associated with tobacco use, the incidence and mortality rate of HPV-induced HNSCC has increased significantly (2). Current treatment modalities for HNSCC include surgery, radiation, chemotherapy, EGFR inhibitors (e.g. Cetuximab) and immunotherapy (e.g. Pembrolizumab) (3, 4). However, the modest improvement in overall survival rates achieved with current therapies emphasizes the significant need for further research in this area.

Salivary gland cancers account for approximately 6% of all head and neck cancers, and present significant treatment challenges due to their rarity and biological diversity (5). Mucoepidermoid carcinoma (MEC) is the most common subtype of salivary gland cancer, followed by adenoid cystic carcinoma (ACC) (6). Conventional chemotherapies are ineffective in salivary gland cancers, and currently no systemic or targeted therapy is approved (7, 8). Given the limited understanding of the underlying pathobiology of these diseases and lack of effective chemotherapeutic approaches, surgery remains the main treatment option for these patients. Considering that both HNSCC and malignant salivary gland cancers follow the cancer stem cell hypothesis, the understanding of the pathobiology of these cells may unveil new therapeutic targets for these tumors.

## Cancer stem cells in head and neck cancer

The traditional, or stochastic, model of carcinogenesis postulates that tumor growth is initiated by a single cell harboring advantageous genetic mutations, which proliferates and dominates the tumor architecture (9). In this model, all subsequently formed tumor cells possess equal potential for tumorigenesis. Nowadays, it is widely accepted that a tumor is highly heterogenous, constituted by cells of varying biological characteristics. The hierarchical model of carcinogenesis suggests that only a unique subset of tumor cells is capable of tumorigenesis, namely cancer stem cells (CSCs) (10). The CSC hypothesis presents that these cells are endowed with the ability to self-renew and give rise to the various cell phenotypes of a heterogeneous tumor through asymmetric and symmetric cell division. CSCs and physiological stem cells share many attributes: the capacity for self-renewal and differentiation, the ability to survive for long periods of time, and strong resistance to harmful agents (11). Hence, the most-accepted hypothesis for the genesis of CSCs remains that they arise from physiological stem cells (12). Other hypotheses include that CSCs arise from physiological differentiated cells or progenitor cells.

Head and neck cancers have been shown to follow the CSC hypothesis and hierarchical model of carcinogenesis, as they are solid, heterogenous tumors consisting of both CSCs capable of tumorigenesis and bulk tumor cells. CSCs have been diligently studied and characterized in multiple types of head and neck cancers, including head and neck squamous cell carcinoma (HNSCC), mucoepidermoid carcinoma (MEC) and adenoid cystic carcinoma (ACC) (13–17). Head and neck CSCs are endowed with properties of invasiveness, quiescence, and epithelial-mesenchymal transition (EMT) – a crucial process in cancer metastasis (18). These cells have been found to reside in perivascular niches, with the majority residing within a 100 µm radius of blood vessels, from which endothelial cell-secreted factors enhance their self-renewal and promote their tumorigenicity (19, 20). This microenvironmental support, along with many other factors, contributes to the increased resistance to therapies observed in CSCs (21, 22).

The CSC hypothesis may explain the resistance to current cytotoxic treatments and propensity for recurrence and metastasis in head and neck squamous cell carcinoma, which are factors that have a negative impact on the long-term survival of these patients. CSCs are resistant to chemotherapies, because these agents generally target cells with high proliferation rates, whereas CSCs proliferate slowly and thus escape their cytotoxicity (23). Therefore, the modest progress of therapies against HNSCC can at least partially be attributed to CSCs, rendering them to be a therapeutic target of interest.

## Biomarkers of cancer stem cells

**Figure 1:**
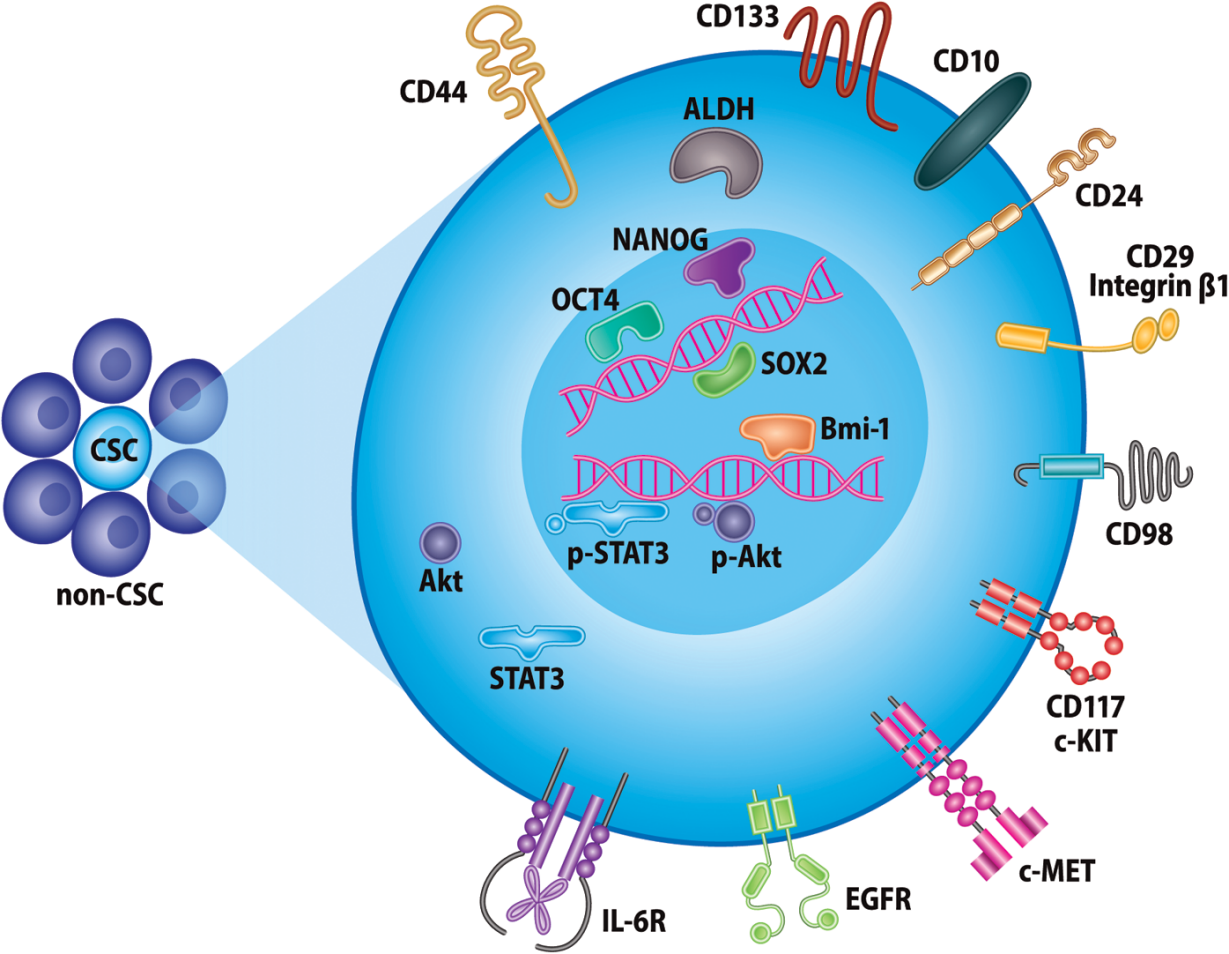
Markers of head and neck cancer stem cells. Selected proteins currently used for identification of CSCs in head and neck cancer. These include, but are not limited to, various cell-surface glycoproteins, receptor tyrosine kinases, intracellular enzymes, and transcription factors.

In order to target CSCs, they must be identifiable by means of unique cellular markers and pathways, which is an area of active research in many cancers. Though differences in CSC biomarkers across cancer types exist, their identification relies heavily on intracellular enzymes, transcription factors, and cell surface molecules. Here, we briefly discuss some commonly used biomarkers of CSCs in head and neck cancer, which are illustrated in Figure 1.

CSCs were first identified in HNSCC as expressing high levels of CD44, a cell-surface glycoprotein for hyaluronic acid that is involved in cell proliferation, survival, adhesion, migration, and intercellular interactions (13). CD44 is one of the most common CSC markers in several malignancies and has been shown to select for highly tumorigenic CSCs as compared to CD44^−^ cells (24). However, since most cells in epithelia of the head and neck express CD44, other biomarkers have been established to refine identification of head and neck CSCs (25). Among these, aldehyde dehydrogenase (ALDH) activity has been accepted as a frequent marker of CSCs. In HNSCC patient-derived xenograft models and cell lines, ALDH^high^ cells demonstrated increased tumorigenicity, therapy resistance, and EMT as compared to ALDH^low^ cells (26–28). As complementary markers, purified CD44^+^ ALDH^high^ cells constitute an even more tumorigenic and invasive cancer cell population, as compared to the other combinations of both markers' expression status (15, 17, 19). Additionally, these cells positively correlate with decreased overall survival, disease grade, and treatment prognosis in patients with HNSCC (29, 30).

CD133, a cell surface glycoprotein, is another prominent yet more debated head and neck CSC marker (31). CD133^+^ cells exhibit increased invasiveness, tumorigenicity, and chemoresistance, but may present only a subpopulation of CSCs in oral squamous cell carcinoma cell lines and tissues (32). CD133 has also been shown to function as a regulatory switch of EMT and stemness properties (33). Multiple other cell surface proteins have been implicated as head and neck CSC markers: CD10 expression correlates with poorer overall survival, local recurrences, and therapeutic resistance (34, 35); CD24^+^ cells promote angiogenesis and tumor progression (36); CD29^+^ cells are highly invasive, migratory, and contribute to metastases (37); CD98^+^ cells are tumorigenic and demonstrate increased expression of DNA repair genes (38).

Various receptor tyrosine kinase (RTK) proteins have also been found to promote chemoresistance, metastasis, and CSC properties in head and neck cancer, with therapeutic targeting of these receptors providing clinical promise. CSCs responsible for Cisplatin-resistance and metastasis have been shown to express high levels of c-Met^+^, and a Phase 1 trial of selective c-MET inhibitor ARQ197 has shown early clinical success (16, 39). The epidermal-growth factor receptor (EGFR) is another example of RTK that is highly expressed in 90% of HNSCC patients and has been linked to treatment resistance, poor clinical outcomes, and higher fraction of CSCs, which, together with CD44, has been shown to promote tumor initiation and progression *in vivo* (40). The EGFR receptor is the target of Cetuximab, an FDA-approved antibody-based therapy currently accepted for treatment of HNSCC (41). The interleukin-6 receptor (IL-6R) is also strongly upregulated in head and neck CSCs and enhances tumorigenicity and self-renewal *via* STAT3 signaling (20). Lastly, CD117 is highly implicated in salivary gland CSCs, where it is also commonly used to isolate progenitor cells of the submandibular gland (42).

Other markers of CSCs are intracellular proteins vital to maintaining stemness such as Oct4, Sox2, Nanog, and Bmi-1. Oct 4, Sox2, and Nanog are markers of pluripotency in embryonic stem cells and crucial for these cells' property of self-renewal (43). CSCs from oral squamous cell carcinoma patient samples exhibited high expression of Oct4 and Nanog, along with CD133, which was correlated with greater tumor stage and worse overall survival prognosis (44). Sox2 expression in head and neck CSCs was responsible for their self-renewal, chemoresistance, invasion, and tumorigenicity *in vitro* and *in vivo* (45). A meta-analysis revealed that Sox2 could be utilized as an unfavorable prognostic factor for higher tumor grade, stage, and metastases (46). Bmi-1 is a polycomb group protein involved in the regulation of normal stem cells. Head and neck CSCs also exhibit high Bmi-1 expression, which has been shown to be required for sphere formation and self-renewal, indicating that Bmi-1 is an important cellular marker for CSC stemness (47). Interestingly, knockdown of Bmi-1 in ALDH^+^ CSCs was shown to also downregulate expression of Oct4, Nanog, Sox2, and c-Myc among other stemness markers in these cells (48). In MEC, intense CD44 and Bmi-1 expression was observed in the tumor invasive front, while Oct4 and Nanog was highly associated with perineural invasion *in vivo* (49).

Many of these cellular proteins have been investigated as potential therapeutic targets for HNSCC, converging in a common denominator of regulating cancer cell stemness (50). In this review, we specifically elaborate on the role and molecular regulatory network of Bmi-1 in mediating head and neck cancer stemness. Additionally, we discuss the current literature on Bmi-1 in promoting therapeutic evasion through chemo- and radioresistance, and the potential therapeutic implications of targeting this master regulator of stemness.

## Bmi-1 and cancer stem cells

### Physiological Bmi-1 function and regulation

Bmi-1 (**B** cell-specific **M**oloney murine leukemia virus **I**ntegration site **1**) is a 37 kDA protein that consists of three domains: N-terminal RING domain, central domain, and C-terminal proline-serine domain. The RING domain at the N-terminal forms a complex with RING1B (51, 52). Bmi-1 is a member of the Polycomb repressive complex 1 (PRC-1) and is involved in H2A-K119 ubiquitination (53) facilitated in part through this interaction between Bmi-1 and RING1B. Polycomb group (PcG) proteins are a family of proteins involved in transcriptional regulation that form complexes such as PRC-1 to facilitate this regulation. The central domain of Bmi-1 contains a ubiquitin-like (UBL) fold that interacts with PHC2, a polyhomeotic protein that is a member of PRC-1; the UBL region also plays a role in the homo-oligomerization of Bmi-1 (54). Lastly, the C-terminal is a proline-serine rich domain that serves as a regulatory domain for Bmi-1 through negative regulation (55).

Bmi-1 plays a direct role in cell cycle regulation and senescence as a negative regulator of the Ink4a locus that encodes p16^Ink4a^ and p19^Arf^, tumor suppressor proteins. Downregulation of Bmi-1 resulted in an increase in p16^Ink4a^ and p19^Arf^ expression leading to senescence, while upregulation of Bmi-1 resulted in a decrease in p16^Ink4a^ and p19^Arf^ expression leading to tumor formation *in vivo* (56). p19^Arf^ is an upstream regulator of p53, a key tumor suppressor protein that functions by blocking MDM2-induced p53 degradation (57). p16^Ink4a^ is an upstream regulator of another tumor suppressor protein: the retinoblastoma (Rb) protein. The phosphorylation of Rb proteins by cyclin D and cyclin E-dependent kinases activates E2F transcription factors, which promotes cellular senescence through entry into the S phase of the cell cycle (58). Thus, Bmi-1 represses two tumor suppressor proteins, p16^Ink4a^ and p19^Arf^, which function by activating senescence and apoptosis respectively.

Bmi-1 has also been implicated in several developmental signaling pathways including Hox, Hedgehog, and Sox2 pathways. The role of Bmi-1 in H2A-K119 ubiquitination has been linked to Hox gene silencing in mouse embryonic fibroblasts when bound to various Hox gene promoters, while Bmi-1 knockdown resulted in de-repression of these genes. This provided evidence that Hox genes are direct targets of Bmi-1-mediated transcriptional regulation and underlines the importance of Bmi-1 in regulation of key developmental processes in progenitor cells (53). Experiments conducted in mammary stem cells illustrated that activating Hedgehog (Hh) signaling upregulated self-renewal and Bmi-1 expression. In these cells within an *in vivo* mouse model, upregulation of Gli1 and Gli2, two downstream transcription factors of the Hh pathway, led to upregulation of Bmi-1 which suggests that Hedgehog signaling mediates stem cell self-renewal through Bmi-1 (59). Bmi-1 is also a downstream target of Sox2, a crucial transcription factor in maintaining stem cell pluripotency and stemness in concert with Wnt signaling. Sox2 inactivation leads to strong Bmi-1 downregulation in osteoblasts *in vivo*, whereas Sox2 overexpression causes Bmi-1 upregulation, and constitutive Bmi-1 expression rescues cell senescence promoted by Sox2 inactivation (60).

Bmi-1 is also regulated by the p38 mitogen-activated protein kinase (MAPK) and Akt pathways. Epidermal growth factor (EGF)-induced Akt activation directly phosphorylates and stabilizes Bmi-1, rendering it resistant to proteasomal degradation and allowing for its nuclear accumulation, whereas p38 inhibits Akt-induced phosphorylation, destabilizing Bmi-1 and promoting increased Bmi-1 degradation in neural stem cells *in vivo* (61). MAPKAP kinase 3 (3pk), a downstream convergence point of p38 and ERK signaling, also regulates Bmi-1 through phosphorylation. 3pK phosphorylation and activation of Bmi-1 resulted in chromatin dissociation and de-repression of Bmi-1 targets, one of which is p14^ARF^ – a tumor suppressor by means of MDM2 inhibition and subsequent p53 stabilization, arresting cells in G1 cell cycle phase (62, 63). As Bmi-1 phosphorylation by 3pk illustrates, the phosphorylation status of Bmi-1 is inversely related to its chromatin association, allowing for fine-tuned regulation of Bmi-1 binding to chromatin throughout the cell cycle (64).

In addition to cell cycle regulation and the maintenance of the stem cell phenotype, Bmi-1 plays a role in reactive oxygen species (ROS) damage and DNA repair. The transcription factor FoxM1c is expressed highly in proliferating cells and was shown to protect them from oxidative stress-induced senescence by directly activating Bmi-1 expression *via* c-Myc *in vitro* and *in vivo* (65). Deletion of c-Myc lead to a decoupling in FoxM1c-induced Bmi-1 expression, emphasizing that c-Myc serves as a bona fide regulatory intermediate in Bmi-1 signaling. Similarly, Mel-18, another polycomb group ring finger protein, downregulates Bmi-1 expression through transcriptional repression of c-Myc in human fibroblasts (66). The absence of Bmi-1 in mice leads to an accumulation of ROS that subsequently triggers the DNA damage response (DDR) pathway (67). The p16^Ink4a^ pathway, which is negatively regulated by Bmi-1, induces ROS accumulation to promote senescence (68). Bmi-1 is necessary for the DDR pathway and is recruited to DNA double-strand breaks, where it contributes to the repair of the DNA lesion with H2A ubiquitination (69). Thus, Bmi-1 contributes to DNA repair through the DDR pathway, but also by preventing elevated levels of ROS in the cell.

**Figure 2:**
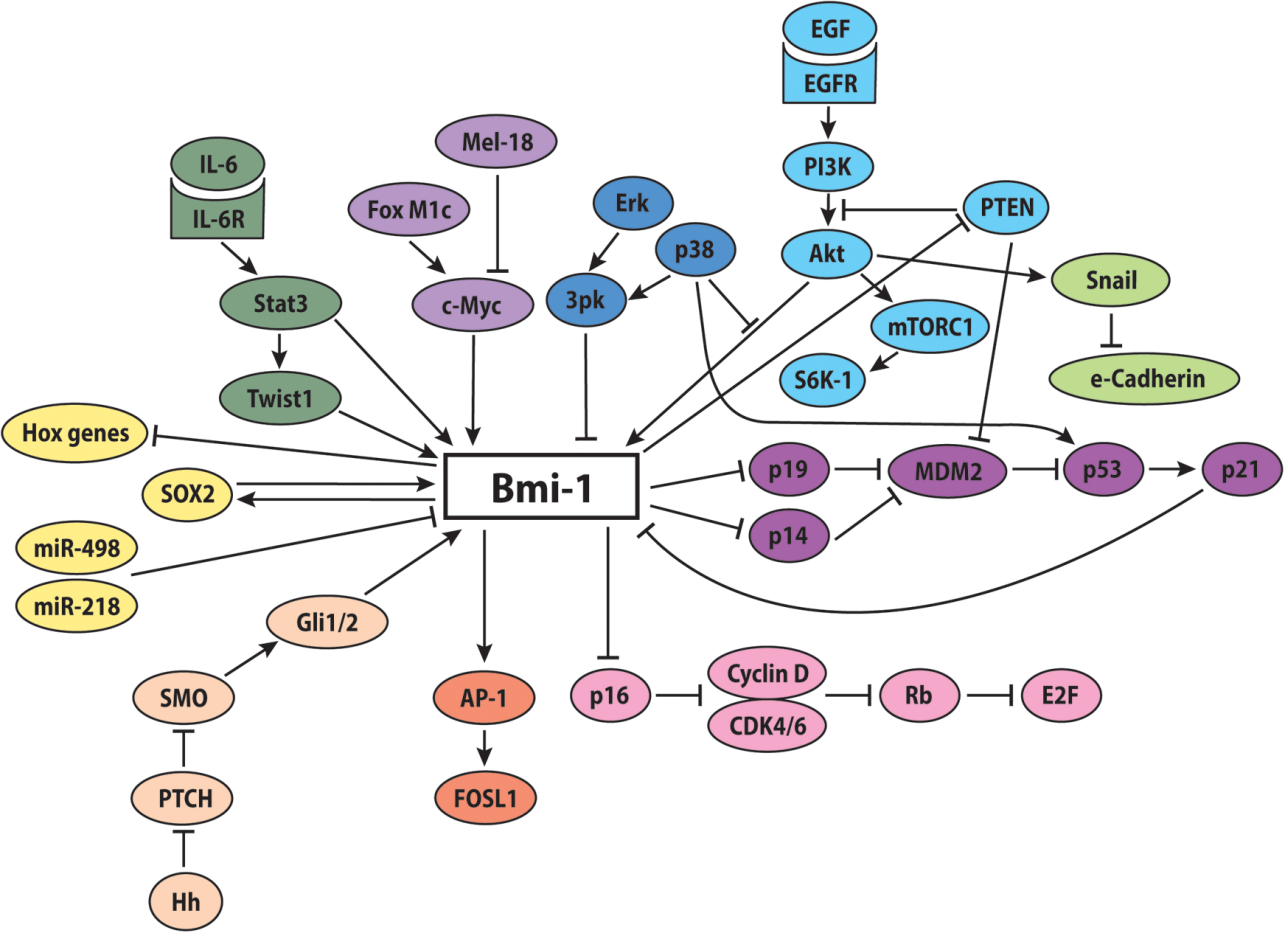
Potential involvement of Bmi-1 in key signaling pathways. The proposed molecular signaling network of Bmi-1 promotes increased stemness, self-renewal, and proliferation, while decreasing apoptosis, differentiation, and senescence.

These reported findings suggest that Bmi-1-mediated repression is a finely regulated and dynamically controlled process, with all arrows pointing to Bmi-1 as a master regulator within the PcG-mediated transcriptional system (Figure 2).

### Bmi-1 regulation in cancer

In head and neck cancer, Bmi-1 is more highly expressed in tumorigenic cells as compared to normal cells. More specifically, elevated Bmi-1 expression is predominantly observed in ALDH^high^CD44^+^ when compared to ALDH^low^CD44^−^ cells *in vitro* and *in vivo* (19, 70), and endothelial cell-secreted factors further induce Bmi-1 expression in the CSCs (19), revealing Bmi-1 as an important player in HNSCC CSC biology. Characterizing Bmi-1 as an oncogene is a novel, active area of research in head and neck cancer, with limited literature on the exact mechanism and signaling pathways in interplay with Bmi-1. Therefore, we will also review Bmi-1-associated signaling pathways in other types of cancer here.

As elaborated above, the Ink4a locus is a direct target of Bmi-1 in normal cells. In breast cancer and oral squamous cell carcinoma, changes in Bmi-1 expression did not affect p16^Ink4a^ expression, suggesting that the oncogenic activity of Bmi-1 functions through a p16^Ink4a^-independent signaling pathway (71, 72). Conversely, in laryngeal cancer, colorectal cancer, gastrointestinal cancer, and non-small cell lung cancer a significant negative correlation between Bmi-1 and Ink4a locus gene expression was observed, suggesting that Bmi-1 promotes cellular renewal through the inhibition of senescence and apoptosis (73–76). In yet another example, Bmi-1-mediated tumorigenesis in liver cancer was not at all related to Ink4a/Arf expression but required for RasV12-driven tumor induction (77). These contrasting findings illuminate the complex dysregulation of Bmi-1 in cancer, highlighting the need for further research in this area.

Another paradoxical relationship has been observed between Bmi-1 and Hox signaling pathways in cancer. Typically, high expression of Bmi-1 results in lower expression of Hox signaling proteins. However, in both Ewing sarcoma and certain leukemias it was observed that despite the expected elevated levels of Bmi-1 expression, there were also elevated levels of Hox expression (78, 79). The underlying mechanism of these surprising findings is not clear, but one hypothesis suggests that this could be due to a mutation that unlinks Bmi-1 and Hox signaling (79) Unlike with Hox signaling, the direct relationship between Hh signaling and Bmi-1 observed in normal cells appears to be maintained in cancer cells. In breast cancer, Gli-1 and Gli-2 overexpression induced Bmi-1 expression, which was necessary to promote self-renewal of both normal and malignant mammary stem cells (59). In ovarian cancer, overexpression of various protein signaling effectors of the Hh pathway also induced Bmi-1 expression (80).

A direct relationship between the Akt pathway regulation of Bmi-1 is also observed in various cancer cells. In MEC, CSCs exhibit constitutive activation of mTOR, Akt, S6K1, and Bmi-1, and it was shown that phosphorylation of S6K1 presents a crucial step in regulation of Bmi-1 *in vitro* and *in vivo* (81). In pancreatic cancer, overexpression of Bmi-1 induced activation of the P13K/Akt pathway by negative regulation of PTEN in CSCs (82). In endometrial cancer, a direct correlation was found between Bmi-1 expression and Akt expression; interestingly, lower levels of both Bmi-1 and the Akt pathway were associated with more aggressive cancer phenotypes, which stands in contrast to most other cancers (83). In gastric cancer, the microRNAs miR-498 and miR-218 inhibited Bmi-1 function, as well as EMT and Akt signaling (84, 85). A similar finding in breast cancer showed that the PcG protein Mel-18 inhibited Bmi-1 and Akt expression, and that constitutively active Akt rescued the tumor-suppressive function of Mel-18 and Bmi-1 inhibition (86). As previously mentioned, Mel-18 suppresses Bmi-1 through inhibition of c-myc in normal cells. This relationship between c-myc and Bmi-1 was supported in a lymphoma mouse model: overexpression of both c-myc and Bmi-1 induced transformation primary embryo fibroblasts (87).

In salivary gland cancer, p53 has been shown to play a central role in regulating the CSC phenotype *via* Bmi-1 (70). Here, it was suggested that p53 reduces CSC stemness not by inducing apoptosis, but rather by regulating Bmi-1 expression *via* downstream p21 signaling and promoting CSC differentiation, and that this mechanism was independent of MDM2. In ACC, therapeutic inhibition of MDM2-p53 was shown to decrease the CSC fraction *via* apoptosis as well as an increase in cells within the G_1_ phase of the cell cycle *in vitro* and *in vivo* (88). Altogether, while Bmi-1 regulation in cancer isn't necessarily conserved as compared to its physiological regulation, these findings highlight Bmi-1 as an important player in tipping the scales between health and disease.

### Bmi-1 in tumorigenesis & metastasis

As previously mentioned, Bmi-1 is highly expressed in head and neck CSCs, which drive tumorigenesis (13), and silencing Bmi-1 leads to a reduction in stemness and tumor formation in HNSCC (48, 47). Bmi-1^+^ CSCs were shown to mediate invasion and lymph node metastases in HNSCC, specifically through increased AP-1 activity and FOSL1 activation, as determined *via* lineage tracing and genetic ablation studies (47). Bmi-1^+^ tumor cells formed significantly more spheres and tumors than Bmi-1^−^ cells, providing strong evidence for the direct role of Bmi-1 in tumorigenesis both *in vitro* and *in vivo*. In MEC, downregulation of p53 promoted tumor growth through expansion of the CSC population and upregulation of Bmi-1, providing evidence for not only the role of Bmi-1 in regulating cancer cell stemness, but also for p53 being a master regulator of cell fate within this context (70). Due to the high rate of metastasis and recurrence observed in head and neck cancer, these findings render the role of Bmi-1 particularly significant and may lead to new therapeutic strategies.

The tumor microenvironment plays a crucial role in supporting cancer cell growth, with microenvironment-associated cytokines and growth factors defining tumorigenic potential of CSCs. In HNSCC, endothelial cell-secreted IL-6 has been shown to promote tumorigenicity of CSCs through STAT3 signaling activation and Bmi-1 expression (89, 20). In fact, endothelial cells were shown to produce a chemotactic gradient through secreted IL-6, which enhances survival and motility of tumorigenic head and neck CSCs (20).

The epithelial-mesenchymal transition (EMT), a process through which a cell shifts from an epithelial phenotype to a more migratory mesenchymal phenotype, is a key feature of invasive cancers and metastases. In nasopharyngeal carcinoma cells, silencing Bmi-1 resulted in a reversal of EMT, exhibited by a shift in epithelial and mesenchymal markers and a reduction in metastases, indicating that Bmi-1 induces EMT resulting in a more migratory and aggressive phenotype *in vitro* (90). This occurs *via* the underlying mechanism of Bmi-1 repressing PTEN, a negative regulator of the PI3K/Akt pathway, which activates this pathway and down-regulates E-cadherin in a Snail-dependent manner. Likewise, upregulating Bmi-1 in ALDH^−^ head and neck CSCs promotes stemness properties, tumorigenicity, and migration by upregulating Snail, an EMT regulatory protein (91). A direct regulatory link has also been established between Bmi-1 and another EMT regulatory protein, Twist1. Twist1 directly binds to the regulatory region of Bmi-1, and both interdependently promote EMT especially under hypoxic conditions (92). Endothelial cell-secreted EGF and IL-6 were also found to induce EMT to enhance the invasive capacity of head and neck CSCs (20, 93). The human telomerase reverse transcriptase catalytic subunit (hTERT) is involved in maintaining the telomeres of cells, thereby prolonging cell life, and also contributes to EMT. Bmi-1 expression mirrored that of hTERT and was required for hTERT-induced EMT marker expression of oral epithelial cells *via* suppression of p16^INK4a^ (94). Altogether, these findings demonstrate the direct role Bmi-1 plays in EMT and therefore, to the more aggressive stem-like and migratory phenotype of tumor cells.

## Bmi-1 in cancer therapeutics

### Bmi-1 in chemoresistance

**Figure 3:**
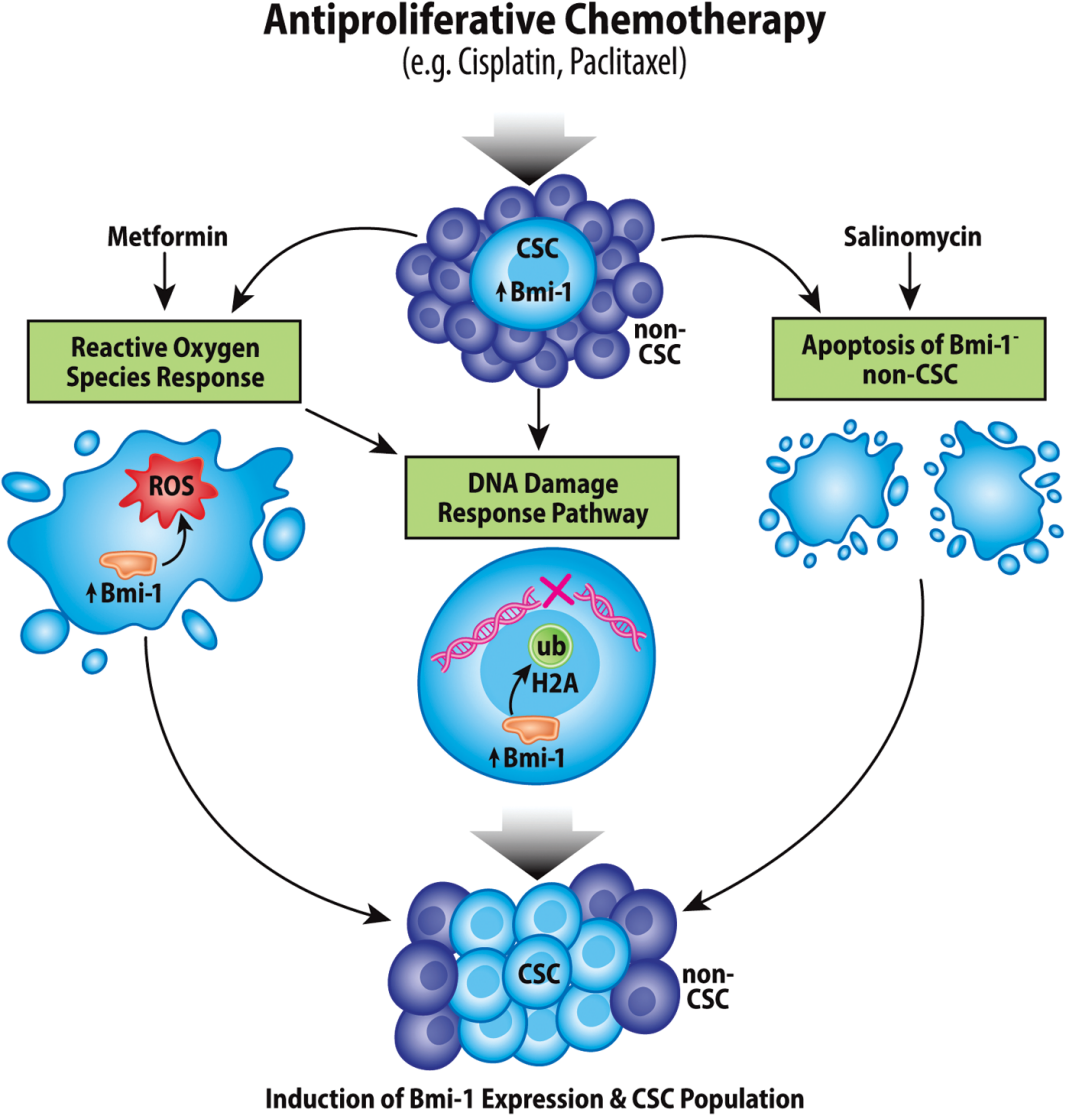
Chemotherapeutic induction of Bmi-1 increases the cancer stem cell population. Antiproliferative chemotherapeutic agents currently used for treatment of head and neck cancers have been shown to increase the CSC fraction in tumors *via* multiple mechanisms, which include inducing the reactive oxygen species response, the DNA damage response pathway, and apoptosis of Bmi-1^−^ CSCs.

The lack of progress in HNSCC, MEC, and ACC treatment can largely be attributed to therapeutic resistance of each of these cancer types, which can lead to both metastasis and recurrence; notably, CSCs are particularly resistant to therapies compared to bulk tumor cells. In the context of head and neck CSCs, Bmi-1 has been strongly implicated in therapy resistance (Figure 3).

Cisplatin is still the most common chemotherapy agent used in treatment of HNSCC, as well as many other cancers. However, it has been shown that treatment with Cisplatin actually increases the CSC fraction in HNSCC tumors, and Cisplatin-resistant cells intrinsically express elevated levels of Bmi-1; there is also a direct association between Cisplatin dosage or resistance and Bmi-1 expression *in vitro* and *in vivo* (22, 95). Interestingly, it has been demonstrated that Cisplatin-induced apoptosis mainly occurred in Bmi-1^−^ tumor cells, and that in HNSCC recurrence specifically Bmi-1^+^ CSC lineages were maintained in these tumors (47). As has been previously mentioned, the IL-6/STAT3 pathway is highly upregulated in head and neck CSCs, and IL-6 augments the Cisplatin-induction of Bmi-1 expression and CSC fraction (22, 89). Inhibition of IL-6 signaling decreased the CSC fraction *in vitro* and *in vivo*, as well as suppressed Cisplatin-induction of Bmi-1 expression and the CSC fraction (20, 22, 95). Similarly, a combination treatment of an AP-1 inhibitor and Cisplatin resulted in inhibition of Bmi-1^+^ CSCs and a reduction in metastases, suggesting a possible mechanistic pathway for chemoresistance *via* Bmi-1 (47). Altogether, these results suggest a clear link between Cisplatin resistance and Bmi-1 expression and support the therapeutic strategy to include the use of either a direct Bmi-1 inhibitor or an inhibitor of a Bmi-1-associated pathway, such as those mentioned above.

Interestingly, Salinomycin, a commonly used antibiotic, successfully targeted CSCs in HNSCC, resulting in reduced Bmi-1 expression and invasive phenotypes of CSCs. When used in combination with Cisplatin or Paclitaxel, Salinomycin greatly increased overall cell death. However, it was found to increase EMT markers, Akt, and mTor signaling, which may correlate with cancer cell stemness (96). As mentioned previously, the PI3K/Akt pathway is highly upregulated in head and neck CSCs and therapeutic inhibition of the mechanistic target of rapamycin (mTOR) has shown clinical success in head and neck cancer (97). mTOR inhibition ablates Cisplatin-induced stemness and blocks Bmi-1 expression in MEC (98). As opposed to in HNSCC, p53 is not oftentimes mutated in salivary gland cancers, and a major therapeutic strategy that has since been translated into clinical trials involves targeting the MDM2-p53 interaction. A small-molecule inhibitor of MDM2-p53 complex (MI-773) has been shown to potently decrease the CSC fraction, Bmi-1 expression, and tumor recurrence in both MEC and ACC (70, 88, 99, 100). Inhibition of MDM2-p53 triggered G_1_ cell cycle arrest, and sensitized tumors to Cisplatin chemotherapy (99, 100). Altogether, these findings illuminate a variety of strategies to overcoming CSC-mediated chemotherapeutic resistance to Cisplatin.

Comparable to the effect of Cisplatin, treatment with Metformin (diabetes drug that has exhibited anticancer properties in various other cancers) lead to a reduction in bulk tumor cells but an increase in CSCs and Bmi-1 expression in HNSCC. Metformin increased expression of Bmi-1, Oct4, Nanog, and CD44, and was revealed to bind to mitochondrial complex III, suggesting a possible role of Metformin in mediating ROS (101). Interestingly, in prostate cancer, Cisplatin functions by elevating intracellular ROS through NADPH oxidase activation (102). Indeed, mechanistic studies revealed that Cisplatin induces a mitochondria-dependent ROS response in conjunction with, but independent from, its well-known cytotoxic effect through inducing DNA damage (103). Therefore, it is hypothesized that many cytotoxic effects of chemotherapeutic agents function through ROS, eliciting protective effects on CSCs and Bmi-1 function. This relates to results described above regarding the role Bmi-1 plays in maintenance of low ROS levels and in the DDR pathway. By elevating ROS and DNA damage levels in the cell, Cisplatin is likely activating Bmi-1 for response to these stimuli, inadvertently activating cancer cell stemness properties.

Thus, further investigation into targeted therapies is necessary, but the treatment for head and neck cancers will likely entail a combination of systemic cytotoxic therapies and CSC-targeted therapies such as small molecule inhibitors of Bmi-1.

### Bmi-1 in radioresistance

In parallel to chemoresistance, radioresistance poses another obstacle in successful treatment of head and neck cancers. In HNSCC, silencing Bmi-1 activity in ALDH^+^ CSCs led to increased apoptotic activity as detected *via* Annexin V staining, resulting in decreased radioresistance and an overall higher survival rate in a mouse model (48). In nasopharyngeal cancer, silencing Bmi-1 resulted in re-sensitization to radiation therapy through increased apoptotic activity of p53 and increased production of ROS (104). These findings implicated Bmi-1 in contributing to HNSCC CSC radioresistance.

Further literature on the role of Bmi-1 in head and neck cancer radioresistance is limited. However, notable findings have been reported in other cancers. Elevated levels of Bmi-1 were also observed in adaptively radioresistant esophageal squamous cell carcinoma (ESCC) cells, where Bmi-1 silencing led to re-sensitization to radiation therapy (105). Similar to the observations made in nasopharyngeal cancer, Bmi-1 conferred adaptive radioresistance to ESCC cells, and Bmi-1-depleted cells treated with radiotherapy expressed elevated levels of ROS and impaired DNA repair capacities, further supporting a common mechanism by which Bmi-1 mediates therapeutic resistance (105). In breast cancer cells, Bmi-1 expression was also indicative of radioresistance. Upon Bmi-1 knockdown, increased DNA double strand breaks, reduced DNA repair, and increased apoptosis through elevated p53, p21 and Bax protein expression was observed (106). In glioblastomas, radiation therapy primarily functions by halting senescence and it was shown that Bmi-1 confers radioresistance by inhibiting cell senescence through the p16 signaling pathway (107). Reduction of Bmi-1 expression by overexpression of microRNA-128 in glioma cells also promoted radiosensitivity and prevalence of senescent cells (108).

Collectively, these studies suggest that Bmi-1 promotes radioresistance through decreased levels of ROS, increased DNA repair, and suppression of senescence through mechanisms resembling those that arbitrate chemoresistance. This also strongly implicates Bmi-1 as a powerful point of convergence in multiple therapeutic resistance mechanisms of different treatment modalities across many cancers.

### Bmi-1 as a prognostic factor

We have reviewed the prominent impact Bmi-1 has on tumorigenesis, metastasis, and therapy resistance of head and neck cancers. Yet, there are many unanswered questions: How does this translate to overall patient survival? How can we leverage this knowledge to better make predictions about patient prognoses and treatment outcomes?

Few research studies to date have robustly investigated Bmi-1 expression patterns in head and neck cancer. Bmi-1 expression in tumor tissue of oropharyngeal squamous cell carcinoma was significantly higher as compared to normal mucosa, but no difference in expression was observed between the primary tumor and lymph node metastases (109). This expression pattern was observed in both HPV-negative and HPV-positive samples (*n* = 12). In another study, HPV-positive human oropharyngeal squamous cell carcinoma specimens showed lower Bmi-1 expression than HPV-negative tumors (*n* = 202) (110). In human tissue specimens, Bmi-1 expression was significantly higher in oral squamous cell carcinoma but showed no difference between normal mucosa and oral dysplasia (*n* = 129) (111). Thus, more elaborate studies are needed to determine the relevance of Bmi-1 expression in head and neck cancer, especially in relation to HPV status and disease progression.

In one study analyzing CSC markers as prognostic factors for HNSCC, it was observed that both Bmi-1 and CD44 are indicators for poorer prognosis of overall and disease-free survival in patients receiving primary radio-chemotherapy irrespective of HPV status (*n* = 85) (112). Conversely, in SCC of the tongue, a strong correlation was observed between low Bmi-1 expression and poor patient prognosis (*n* = 73) (113), and in a separate meta-analysis (*n* = 2143), Bmi-1 did not impact overall HNSCC survival significantly (114). In another study, Bmi-1 expression in patient samples (*n* = 216) was correlated with poor prognosis of recurrence-free survival, but not overall survival (95). These observations may indeed be explained by the CSC hypothesis, since the small CSC fraction may not significantly contribute to overall tumor growth and survival, but to tumor recurrence or metastases. This ambiguity illustrates a definitive need for further research on Bmi-1 as a prognostic factor in HNSCC.

In other cancers, Bmi-1 has been a more promising negative prognosticator. Bmi-1 overexpression has been reported in a plethora of cancers, including gastric, ovarian, breast, head and neck, pancreatic, lung, hepatocellular, and endometrial carcinoma and correlated with a variety of indicators of poor prognoses as described elsewhere (82). A meta-analysis of non-small cell lung cancer revealed a correlation between elevated Bmi-1 expression and increased tumor size, metastasis, and lower overall survival rates (115). Bmi-1 was also found to be a negative prognostic factor in gastric cancer and endometrial adenocarcinoma, each demonstrating heightened Bmi-1 expression to be indicative of worse clinical stage, lymph node metastases, and overall survival (116, 117). Based on these varied and contradictory findings, the utility of Bmi-1 as a prognostic factor remains unclear. One plausible explanation for this may be the lack of expression analyses specific to a tumor subsite or clonal cell population. As mentioned previously, tumor cells as well as a plethora of stromal cells from the tumor microenvironment express Bmi-1, which highly clouds the prognostic value of this marker. While these studies do not yet provide convincing evidence for Bmi-1 serving as a possible way to prognosticate treatment response, Bmi-1 is undoubtedly an important master regulator of cancer cell stemness and therapeutic resistance, rendering it a putative therapeutic target, nonetheless.

### Therapeutic targeting of Bmi-1

Bmi-1 has emerged as an attractive therapeutic target in CSC-focused, mechanism-based cancer treatments. Therapeutic inhibition of Bmi-1 was first described in a primary colorectal cancer xenograft model, where it inhibited CSC self-renewal and thus abrogated their tumorigenic potential (118). The anti-PD-1 immunotherapies Nivolumab and Pembrolizumab have been approved as first-line therapies for HNSCC and are commonly combined with Cisplatin treatment. In an *in vivo* mouse model of HNSCC, Bmi-1^+^ CSCs were enriched in tumors following treatment with Cisplatin and anti-PD-1 therapy, but treatment with the Bmi-1 inhibitor PTC209 prevented induction of these cells and tumor progression (119). In this study, Bmi-1 inhibition was also shown to promote CD8^+^ T-cell infiltration by removing repressive H2A ubiquitination to induce transcription of chemokines necessary for their recruitment. Interestingly, Bmi-1 may play a significant role as immune modifier in several *in vivo* studies: Bmi-1 inhibition restored B-cell-mediated humoral immune responses *via* increased antibody function (120). Immune escape of pancreatic cancer cells from NK cell-mediated elimination in a hyperglycemic tumor microenvironment was also shown to be mediated by upregulation of Bmi-1 and subsequent MICA/B inhibition and GATA2 promotion (121). In a murine myeloma model, Bmi-1 inhibition eliminated tumor-associated macrophages and mediated chemoresistance (122).

The therapeutic potential of Bmi-1 inhibitors has also been investigated in early human clinical trials. In a Phase 1 multi-center, open-label study in patients with advanced solid tumors, the second-generation Bmi-1 inhibitor PTC596 was determined to be tolerable with manageable side effects (123). Notably, PTC596 was shown to be successful in the treatment of acute leukemia in an *in vitro* study irrespective of p53 mutational status, which could provide highly beneficial in the treatment of salivary gland cancers which typically demonstrate high mutational burden of p53 (124). Another Bmi-1 inhibitor, PRT4165, prevents accumulation of all detectable H2A ubiquitination sites around DNA double-stranded breaks in an osteosarcoma model, which could be highly relevant in combination with antiproliferative chemotherapies that propagate the DNA damage response as mentioned in Figure 3 (125). In an ovarian cancer model, the Bmi-1 inhibitor PTC028 was shown to selectively inhibit cancer cell growth while leaving normal cells unaffected, which could present a unique benefit as compared to conventional chemotherapeutic agents that do not selectively eliminate tumor cells (126). While there are limited published studies on the efficacy of Bmi-1 inhibitors in head and neck cancers, the success of these small molecule drugs in treatment of other cancers in preclinical and clinical models strongly supports their therapeutic potential.

## Conclusion

There has been modest progress in the outcome of head and neck cancer patients, in part due to therapy resistance which can be attributed to the function of CSCs in tumorigenesis and tumor dissemination. This emphasizes the need for further research into the underlying mechanisms regulating the CSC phenotype. Bmi-1, a polycomb complex protein, has been established as a pivotal player in controlling cancer cell stemness. It has been implicated in many major signaling pathways such as the p16^Ink4a^/p19^Arf^, PI3K/Akt, MAPK, STAT3, and Hedgehog pathways. It also plays vital roles in cellular processes responding to ROS and DNA damage. In cancer, it has been widely linked to increased stemness, tumor formation and metastasis, as well as therapeutic resistance. This review attempted to synthesize the current evidence on Bmi-1 within the context of head and neck cancer stem cells, and to provide support for future research aimed at targeting this master regulator of cancer cell stemness using novel therapeutic approaches.
